# The neurobiology of play: a narrative review of evidence from mice and humans for advancing neurorehabilitation

**DOI:** 10.3389/fnins.2025.1729411

**Published:** 2026-01-06

**Authors:** M. E. Canepa, L. A. Ramenghi

**Affiliations:** 1 Department of Neuroscience, Ophthalmology, Genetics and Mother-Child Health, University of Genoa, Genoa, Italy; 2 IRCCS Giannina Gaslini, Genoa, Italy

**Keywords:** aging, brain, child, development, neuroplasticity, play, rat, social behaviour

## Abstract

**Objective:**

This narrative conceptual review explores the neurobiological underpinnings of play behaviour across species, with an emphasis on how play affects brain development, social functioning, and cognitive outcomes from early life through aging.

**Methods:**

We synthesize current neuroscientific literature from animal and human studies, focusing on translational evidence involving specific brain regions (e.g., prefrontal cortex, amygdala, striatum), neurochemical systems (e.g., dopamine, opioids), and behavioral domains (e.g., executive function, emotional regulation, and social cognition). Studies are categorized by developmental stage and functional impact.

**Results:**

Evidence from rodent models demonstrates the activation of distinct neural circuits during structured and spontaneous play (e.g., hide-and-seek, rough-and-tumble), with sex-specific differences in cortical and subcortical engagement. In humans, play emerges in infancy and supports neural plasticity, language development, and executive functioning. Later in life, playfulness correlates with cognitive resilience and may act as a protective factor against neurodegeneration. The review also highlights play-based rehabilitation approaches (e.g., sensory-motor therapy, LEGO®-based interventions, sports) with demonstrated neurological and psychosocial benefits.

**Conclusion:**

Play is a multidimensional, evolutionarily conserved behaviour that engages neurobiological systems critical to development and health. Although promising evidence supports play-based interventions, further research is needed to clarify mechanisms, optimize therapeutic use, and bridge species-specific findings in translational neuroscience.

## 1 Introduction

Play is often associated with early childhood, symbolized by kindergartens, toys, and creative expression. Indeed, play-based rehabilitation techniques—such as art therapy—are widely recognized for their benefits in paediatric and neonatal care, supporting cognitive, motor, and emotional development. However, this conceptual narrative review intentionally shifts away from such established domains to explore a more fundamental question: when does play begin, and how does it shape the brain?

This inquiry invites a cross-species exploration of play as a neurobiological phenomenon—one that emerges early in life and contributes to lifelong cognitive and social functioning. Frequently, rodent evidence is previously verified and collected before experimenting on human samples as translational neuroscience does. Through a neuroscientific lens, we will examine the ontogeny and neurodynamics of play in animal and human models, highlighting how early play behaviours reflect—and shape—key brain circuits involved in executive function, emotional regulation, and social cognition.

The discussion is structured into four sections. The first section analyses animal models, particularly rodent play behaviour and its neural substrates, with a focus on timing, motivation, and sex-based differences. The second explores human developmental trajectories, tracing the onset of play from infancy to adulthood and examining how play contributes to neurodevelopment and psychological wellbeing across the lifespan. Thereafter, the third section analyses some examples of play-based rehabilitation therapies (LEGO-based therapy, sports and sensory-motor playing). Last, a discussion section is proposed on the convergences and current gaps of literature.

## 2 Playing effects in neuroscientific literature: cognitive, social and behavioral dimensions

The current neurobiological and rehabilitation evidence is summarized in Table 1 and explained in detail in the paragraphs below focusing on mice per activity (2.1.) and humans per age (2.2.), whereas section 3 is dedicated to neurorehabilitation play-based strategies (sensory-motor playing; LEGO-therapy; sports).

**Table 1:** Current evidence of neurobiological and rehabilitation literature on playing (mice-humans).

Play type	Species	Neural systems involved	Key outcomes
Hide-and-seek	Rats (Murine)	Prefrontal cortex (PFC)	PFC engaged during active play; distinct neural states related to role play and phases of game (Reinhold et al., 2019; Bagi et al., 2022).
Rough-and-tumble	Rats	Prefrontal cortex, amygdala, dopamine system, 50-kHz USV pathways (Cacna1c gene impact)	Sex differences in play vigor and vocalization; important for social behaviour and stress coping (VanRyzin et al., 2020; Kisko et al., 2021).
Social play	Rats, Hamsters	Thalamic intralaminar nuclei, striatum, amygdala, hypothalamus, dopamine/opioid/cannabinoid systems	Social play supports emotional regulation, social bonding, and is modulated by neurochemistry (Siviy and Panksepp, 2011).
Neonatal handling	Rats	Motivation-related systems, possibly dopamine and tactile sensory processing circuits	Neonatal handling increases play motivation and positive social emotions like joy (Aguilar et al., 2009).
Sensorimotor play	Humans (Infants)	Motor circuits, sensory-motor integration pathways	Promotes neuromotor development; handling fosters exploration and agency (Håkstad et al., 2017; Harbourne et al., 2021).
Symbolic/pretend play	Humans (Toddlers)	Prefrontal cortex, language circuits	Facilitates language acquisition and executive function; seen from age 2 + (Scott and Cogburn, 2023).
Playful interaction	Humans (Children)	Executive function circuits (PFC), limbic system	Improves attention, mood, and social bonding (Yaffe et al., 2025; Duss et al., 2024).
LEGO-based therapy	Humans (Children)	Orbitomedial, anterior PFC, dorsolateral PFC, memory systems	Enhances executive function and memory in children with ASD, epilepsy, CHD (Zaldumbide-Alcocer et al., 2024; Espinosa-Garamendi et al., 2022).
Team-based sports	Humans (Children)	Cortical circuits supporting motor planning, cognition, and social engagement	Team sports more effective than sport solo activity in boosting executive function (Yang et al., 2024).
Social playfulness	Humans (Elderly)	Global brain networks; hypothesis-driven links to PFC and hippocampus	Positively associated with cognitive resilience; potential protective role in aging (Golland et al., 2025; Proyer, 2014).

### 2.1 Animal models

#### 2.1.1 Hide-and-seek

In murine models, prefrontal cortex was involved in active hide-and-seek playing. Reinhold et al. (2019) found on *Science* that “when hiding, rats vocalized infrequently and they preferred opaque over transparent hiding enclosures, a preference not observed during seeking. Neuronal recordings revealed intense prefrontal cortex activity that varied with game events and trial types (“hide” versus “seek”) and might instruct role play.”

Based on these results, Concha-Miranda et al., 2020 studied juvenile male rats in a 12:12 h inverted light/dark cycle with free access to food and water ad libitum. As described by the authors, mice were housed individually to increase their social bond to the experimenter as their sole interaction partner. Experimental manipulations started at postnatal day 21 for all animals. For habituation, training and experiments, animals were brought into the 5 m × 4 m illuminated room; animals adapted quickly to the room and the illumination (100–140 lux). Three hiding places for the experimenter were built using large cardboard boxes and positioned in equal distance to the center of the room. As additional hiding locations for the animals, four boxes were provided with an opening made from transparent plastic; two boxes were sprayed opaque. To make the boxes more attractive, a small piece of cloth was included. In this study, prefrontal cortex wasn’t engaged during playing observation (Concha-Miranda et al., 2020). To more clarify this phenomenon, Bagi et al. (2022) proposed an alternative approach characterized by a more naturalistic and less human-forced experimentation setting. Specifically, they explored neural population activity in the prefrontal cortex (PFC) of rats playing hide-and-seek with a limited number of simultaneously recorded cells (≤ 31) able to reflect aspects of the task what aspects of the task. Based on hidden Markov models, population activity in the PFC was clustered into a set of neural states, each associated with a pattern of neural activity. Despite high variability in behaviour, relating the inferred states to the events of the hide-and-seek game reveals neural states that consistently appear at the same phases of the game. Indeed, they found out that prefrontal cortex was “in distinct sets of states” in both playing and observation.

#### 2.1.2 Rough-and-tumble: the model system to study playfulness in relation with social behaviour and sex differences

However, playing in these animals is also an interesting item to study the so-called “social brain,” that is, neurological dynamics pertaining to social behaviour. Indeed, Siviy and Panksepp (2011) defined the rough-and-tumble “as a model system to study playfulness” according to the supporting international data. As this playing social behaviour is highly conserved along mammals and male rats engage in more frequent and vigorous play than female rats, Jonathan VanRyzin et al. proposed in 2020 a protocol of a behavioral testing paradigm to assess social play in male and female juvenile rats with behaviour scoring criteria for distinguishing rough-and-tumble play from other play-related social behaviours (VanRyzin et al., 2020). These sex differences are supported by international research. In a spectrographic analysis on sex differences in the acoustic features of social play-induced 50-kHz ultrasonic vocalizations (USV) in wild-type Sprague–Dawley and Cacna1c haploinsufficient rats (Kisko et al., 2021), not only juvenile male rats engaged more loudly in tough-and-tumble (50-kHz USV) than quieter female rats (<50-khZ USV), but Cacna1c gene haploinsufficiency affected the emission of 50-kHz USV during rough-and-tumble play in female rats. These authors provided evidence that such effects of Cacna1c haploinsufficiency were conducted by male-typical features of 50-kHz USV emission, and louder vocalizations mirrored the “rewarding nature of play and serve as socio-affective signals” during playing. When analyzing sex differentiation of fighting even in terms of the motivation to initiate play, female and male rats are characterized by a diverse interaction between the sensory capacity to detect and respond to a play partner, the organization of the motor patterns used to interact with a partner, age-related changes at puberty in initiating play and in responding to playful contact, and dominance-related changes in adulthood in the pattern of playful interaction (Pellis et al., 1997) because some of these mechanisms are sex-typical and not play typical involving quantitative and qualitative differences (Pellis et al., 1997). These sex differences pertain to neural circuits disruptions regulating aggression in play deprivation in other rodent animals, such as hamsters (Kyle et al., 2019). Play deprivation incremented fighting back during social defeat stress in males, while in females it reduced aggressive behaviour during conditioned defeat testing. However, similar effects in male and female hamsters were observed on neural circuits regulating stress responsivity (Kyle et al., 2019). So, juvenile social play functions foster coping with stress and appropriate social behaviour in adulthood because play deprived-hamsters were characterized by defeat-induced social avoidance independently of sex (Kyle et al., 2019). To give more solidness to the social behaviour connected with playing, Pellis and colleagues studied juvenile rats while playing rough-and-tumble (Pellis and Pellis, 1987; Pellis et al., 2022).

In tough-and-tumble, mice have individual play styles and partner preferences (Ham and Pellis, 2024) and they balance familiarity and novelty in social play preferences (Ham and Pellis, 2023). Even in play partners choosing, male rats preferred ones engaging in more turn taking and with partners with whom they had more symmetrical play relationships (Ham and Pellis, 2025). Noteworthy, fighting with peers seemed to be crucial also for the development of executive functions (Pellis et al., 2017; Pellis and Pellis, 2017).

#### 2.1.3 Social play behaviour: a multidimensional phenomenon

To deeply dive into social play behaviour in mice, international research strived to find out different substrates of this complex phenomenon.

Accordingly, Siviy and Panksepp (2011) tried to summarize neurobiological substrates of social play behaviour in mammals’ brain according to international research. They defined play as “a fundamental and intrinsic neurobehavioral process” splitable into two dimensions:

1. Neurobehavioural sphere. The thalamic intralaminar nuclei, frontal cortex and striatum constitute a primary-process executive circuit for play in the rat. It may interact with other neural areas like amygdala, ventral hypothalamus, periaqueductal gray (PAG), deep tectum and ascending dopamine systems.
2. Neurochemical sphere. Specific cholinergic and dopaminergic controls, endogenous opioids and cannabinoids modulate positively playfulness, with all neuropeptides known to have aversive effects to reduce play. Monoamines such as norepinephrine and serotonin certainly modulate play, but they influence all psychobehavioral systems.

#### 2.1.4 Neonatal handling stimulation

Spanish neuroscientists (Aguilar et al., 2009) aimed to “establish whether the range of effects of neonatal handling stimulation (H), that is, brief daily periods of infant isolation, could be extended to the domain of social motivation. With this aim, the authors studied the innate motivation to engage in rough-and-tumble play (R&T) in adolescent rats (*Rattus norvegicus*) by means of a reversal design, in which half of the rats were first housed in isolation (Days 1–3), and then in company (Days 4–6), while the other half followed the reverse sequence of housing conditions. Results showed in a clear-cut manner that H fuelled playfulness, as measured by pin and dorsal contact episodes, with (relative) independence of trait-based differences in fearful behaviour between handled and nonhandled rats. Given that the different levels of the rat’s social brain are apparently sensitive to tactile stimulation in infancy, the authors propose that the vibrant R&T reported here could reflect an enduring alteration of genetically based, motivational systems underlying playfulness and, perhaps, positive social emotions like joy.”

In sum, playing is crucial for animal neurobehaviour and social skills. Thanks to neuroscience studies, researchers will be able to explore more deeply these aspects which are important even in human studies as reported in the following section organized by age (neonates and infants; toddlers and children; adolescents; elderly).

### 2.2 Human models

#### 2.2.1 Neonates and infants

Before exploring neurological effects of playing in humans, a pivot is to establish at which age play begins. Scott and Cogburn (2023) explained it very well: “Play is prosocial and evolves as the child progresses through infancy and childhood. The earliest and most rudimentary indication of play is the social smile, which occurs at about 4–6 weeks of age. By 3 months, play progresses to regular smiling and cooing when a baby is face-to-face with a person. Between the ages of 3–6 months, infants actively seek interaction with others and begin to play “baby games” like peek-a-boo with their caregivers. At this milestone, children begin using their caregiver for enjoyment in addition to comfort and security. Play at this stage is repetitive and often ranges in intensity according to how the baby responds to the game. Play starts “primitively” as sensorimotor play from 1 to 2 years with an exploration of properties and functions of objects. During this age, pretend play is initiated with the imitation of ordinary activities like pretending to eat or imitating caregivers. Between the ages of 1 and 2, imaginative or pretend play replaces sensorimotor play. Pretend play is a direct imitation of ordinary activities. Pretend play extends to symbolic play, which is the use of one object for another. Symbolic play can be a vehicle for relief from reality. By the age of 2, a toddler’s engagement with others will extend to peers their same age, not just their caregivers.” This phenomenon is quite similar to what we saw in rats.

#### 2.2.2 Toddlers and children

Siviy and Panksepp (2011) described playing as crucial for childhood neurodevelopment in rodent models. This is comparable with human studies focusing on paediatric cohorts. Indeed, the international longitudinal study by Waldman-Levi et al. (2022) stated that cognition mediates playfulness in early child age in cohorts of children at 6, 18 and 24 months of age. In Switzerland, Duss et al. (2024) analysed children playfulness with parents over 2 years. They noticed that “repeated-measures hierarchical linear models indicated significantly lower levels of playfulness in the children with low executive functions (EFs) than in the controls, with no significant changes observed over 2 years in either group. In the children with low EFs, we found a significant positive relationship between parental playfulness at T1 and children’s playfulness 2 years later but a significant negative relationship between parental playtime at T1 and children’s playfulness 2 years later. These results prompt a broad discussion on potential implications for the enhancement of playfulness in children with low EFs within the family environment.” Specifically, maternal playfulness highly influences children development of language, problem-solving and personal-social skills when mothers were not too intrusive in their offspring playing (Léniz-Maturana et al., 2023). Besides family contexts, Yaffe et al. (2025) demonstrated on *Scientific Reports* that “playful interaction, but not physical activity, improved attentional performance, and in particular response times in the Flanker task. Additionally, playful interaction enhanced children’s positive mood and led to stronger social bonds with the co-player. These promising findings suggest that playful interactions are multidimensional activities that simultaneously engage cognitive, emotional, and social functions. We suggest that social playfulness holds unique potential for interventions aimed at training EFs in primary school children, as it is highly enjoyable and easy to learn and integrate into daily activities.”

#### 2.2.3 Adolescents and adults

Neurologically, human prefrontal cortex is probably activated and benefitted during ludic activities together with striatum and amygdala (Siviy, 2016) and moreover, like rats (Vanderschuren et al., 2016), human adolescents’ behaviours are reward-linked processes during positively enriched experiences inducing neurobehavioral plasticity via hippocampus (Fernández-Teruel, 2021). According to Siviy (2016), play is not an indispensable component like feeding and drinking. Nonetheless, playing before puberty “gives an added edge over those they do not play.” That’s why cognition and cognitive health are important to be mentioned as play is associated with them. How much is playfulness crucial for all-age health? In an online survey study in 2014 (Proyer, 2014) involving 4,100 adults, “playfulness seems to be of relevance in all age groups and displays robust relations with different indicators of wellbeing” like a pleasurable life and happiness.

#### 2.2.4 Elderly

Even in aging, social playfulness may be potentially pivot as a neuroprotective strategy against cognitive decline, although further research is needed to prove it. However, international researchers proposed brilliant projects and frameworks. For example, Golland et al. (2025) on *Frontiers in Neuroscience* reviewed the on-topic literature and thereafter, they proposed a multidisciplinary framework combining neuroscience with cognitive psychology, and creative-arts therapies in order to provide a powerful tool to turn the hypothesis of social playfulness as cognitively beneficial into evidence. Accordingly, the following section is centered on playing as a neuro-beneficial rehabilitation technique according to the current evidence.

## 3 Some evidence-based examples of playing as a neuro-beneficial rehabilitation technique

International literature has proved the positive outcomes on brain and nervous system while playing in different categories of patients in pediatrics, adult medicine and geriatrics.

### 3.1 Sensory-motor playing

Preterm birth is one of the main causes of brain injury leading to motor and cognitive impairments. As a matter of fact, the first rehabilitation technique to implement is the so-called “sensory-motor playing.” Generally, physiotherapists (PT) are the main care providers to improve infant’s motor skills. In a 2017 Norwegian multicentric observational study involving infants at 3–14 months of age born prematurely at gestational age (GA) ≤ 33 weeks (Håkstad et al., 2017), all physical therapy sessions were with one or both parents present, either in the family’s home or at the physiotherapist’s workplace. Floor space was an available and natural site for the conduction of physical therapy. The infants quickly adapted to the researcher’s presence. The PTs and parents were encouraged to proceed with the session as usual and not make changes to accommodate the researcher.

Each triad received three visits over a 5–10 months period, amounting to 20 physical therapy session observations (due to cessation of physical therapy, one triad received only two visits). Therapeutic handling could improve infant’s motor performance and enable their discovery and pursuance of new sensory-motor play possibilities. Handling enabled their detection of the infant’s directional movements, use of force and changes in muscle tone; all of which informed PTs about the infant’s engagement, compliance and capacity during the play activity. This study underscores the sensory-motor rehabilitation in the context of care personalization as physiotherapists have to “plan and tailor the intervention to match the infant’s interests; attune themselves to the infant’s intentions; and incorporate therapeutic measures in sensory-motor play interactions with the child.” Plus, in a multisite randomized trial involving 111 infants with neuromotor disorders and submitted to a specific neurorehabilitation program named “START-Play” (Sitting Together And Reaching To play) (Harbourne et al., 2021), play opportunities with the PT professional may foster motor response and cognitive problem-solving using Bayley scales to assess neurodevelopmental outcomes (motor, cognitive and social items). Even analyzing cohorts of elder children, the sensory-motor play can be integrated with the current rehabilitation plan to enhance the sense-of-agency and refine the range of possible movements (Håkstad et al., 2022) based on the beautiful metaphor used by Rosenbaum (2014) to describe as jungles the abundance of brain connections in children. In this context, play is a manner to explore this jungle of neurons and synapses (Sutton-Smith, 1997) managing to select and consolidate the most useful connections for the child (Hadders-Algra, 2018).

### 3.2 Analogic playing (“offline”): the LEGO lesson

Furthermore, LEGO bricks are used as a rehabilitative therapy to improve both social behaviour and neural function in terms of activating programming and organizational skills in relation with memory and problem-solving in several diseases (Vegni et al., 2023) like ASD (Autism Spectrum Disorders), epilepsy and other conditions. For instance, in a 2024 study on epileptic children (Zaldumbide-Alcocer et al., 2024) with ages ranging around 10.33 ± 2.96 and 11.9 ± 3.17 years for the LEGO® B-T and control groups without LEGO B-T treatment, cognitive BANFE-2 scores increased significantly in the orbitomedial, anterior prefrontal, and dorsolateral areas within LEGO B-T group compared with the control group. Moreover, in the gain score analysis, the orbitomedial and memory scores were significantly different from the control group. These results are comparable with another study on children with congenital heart diseases (>50% of children suffered from cognitive impairment), BANFE scores significantly incremented in the brain areas related to executive functions within patients submitted to LEGO-based therapy (Espinosa-Garamendi et al., 2022). The literature is more illustrative concerning ASD (Autism Spectrum Disorders) patients, especially related to the benefits concerning social behaviour within ASD children and young adults, like adaptive, prosocial and communicational behaviours even in primary and secondary education (Wright et al., 2023; Barr et al., 2022; Lindsay et al., 2017; Narzisi et al., 2021; Angelis et al., 2024). Indeed, Vegni et al. (2023) found on an ASD child “notable improvements on the working memory subtest by 1 standard deviation and on speed processing by 2 standard deviations. This (improvement of memory and executive function, ndr) is also evident in Vincent’s (name of the involved child) performance on the Tower of London test, which specifically evaluates executive function (EF). Prior to training, Vincent’s performance was so low that it could not be assessed. However, following training, Vincent demonstrated measurable progress and was able to complete the task, albeit with some difficulty.” Particularly, this group encouraged researchers to explore more deeply the potentialities of this LEGO-Therapy in improving children’s cognitive, social and communicative skills. For aging diseases, the copyrighted project “Bricks for Better Brains” included bright-colored LEGO as one of the main pillars to enhance cognitive skills and engagement in elderly with dementia (Kasperovich and Sasser, 2024). Choosing another famous jigsaw (Tetris), scholars identified the so-called “Tetris effect” (Jassim et al., 2025) as the implicit drive for perceptual cohesion both in autistic and neurotypical patients with logical combination from separate pieces.

### 3.3 Sports

Sports like judo could enhance central nervous system maturation in preschool children according to a multinational, mixed-methods and follow-up study on *Frontiers in Psychology* (Križalkovičová et al., 2024). Moreover, judo could foster the maintaining or reducing body fat, increasing bone mineralization, and improving the function of the cardiorespiratory system compared to the non-practicing control group within children and adolescents (Kowalczyk et al., 2023). These results are coherent with previous child rehabilitation research inviting to frame optimal sport activities participation in order to reach psychological, cognitive, perceptual and neuromotor maturation milestones depending on every developmental age (from infancy to 11 years old) (Patel et al., 2017). It is important to consider which kind of sport is more beneficial. Indeed, a further cohort study on 880 children, “moderate to vigorous physical activity at ages 5–6 years was not associated with executive function at ages 10–11 years. Compared with participants in individual sports, children involved in team sports or both types of sports exhibited superior executive function at ages 10–11 years” (Yang et al., 2024).

## 4 Discussion

The international research showed playing as a multidimensional activity involving cognitive, social and neurobiology aspects. In animal and human models, playing is neuro-beneficial across lifespan and these benefices are shaped continuously according to sex, playing partners (parents/peers) and other covariates. Although it astonishingly begins in neonatal age with increasingly social, cognitive, behavioral and emotional skills, very few was done to balance the number of animal and human studies, where rodent-based models are more spread than humans-based ones.

This conceptual narrative review aimed to be a little and not-exhaustive handbook to comprehend the importance of playing based on the current neurobiology evidence and gaps. This kind of literature could help researchers and clinicians for better investigating on the most fitting play-based neurorehabilitation techniques able to enhance neurological outcomes, social behaviour and cognition. Neuroscientific research should further investigate more on these aspects to revolutionize rehabilitation in a more brain-stimulating way. For instance, they should focus on setting up concrete and feasible initiatives based on a trade-off between current evidence, available resources, realistic limitations. The proved gaps like small size of samples, absence of patient category per age (elderly, children, etc.) and play activity as well as heterogenous results in the literature may be overwhelmed keeping track of the most solid scientific background and methodology together with considering the pitfalls and potentials of current analysis and report tools.

## Data Availability

The original contributions presented in the study are included in the article/supplementary material, further inquiries can be directed to the corresponding author.
